# Therapeutic glucocorticoids prevent bone loss but drive muscle wasting when administered in chronic polyarthritis

**DOI:** 10.1186/s13075-019-1962-3

**Published:** 2019-08-01

**Authors:** C. G. Fenton, J. M. Webster, C. S. Martin, S. Fareed, C. Wehmeyer, H. Mackie, R. Jones, A. P. Seabright, J. W. Lewis, Y. C. Lai, C. S. Goodyear, S. W Jones, M. S. Cooper, G. G. Lavery, R. Langen, K. Raza, R. S. Hardy

**Affiliations:** 1 Institute of Inflammation and Ageing, University of Birmingham, Birmingham, UK; 2 Institute of Metabolism and Systems Research, University of Birmingham, Birmingham, UK; 3 MRC Arthritis Research UK Centre for Musculoskeletal Ageing Research, University of Birmingham, Birmingham, UK; 4 Centre for Endocrinology, Diabetes and Metabolism, Birmingham Health Partners, Birmingham, UK; 5 ANZAC Research Institute, University of Sydney, Sydney, Australia; 6 Sandwell and West Birmingham Hospitals NHS Trust, Birmingham, UK; 7 Centre of Immunobiology, Institute of Infection, Immunity and Inflammation, College of Medical, Veterinary and Life Sciences, University of Glasgow, Glasgow, UK; 8 Department of Respiratory Medicine, NUTRIM School of Nutrition and Translational Research in Metabolism, Faculty of Health, Medicine and Life Sciences, Maastricht University, Maastricht, Netherlands; 9 School of Sport, Exercise and Rehabilitation Sciences, University of Birmingham, Birmingham, UK; 10 Institute of Clinical Sciences, University of Birmingham, Birmingham, UK

**Keywords:** Polyarthritis, Glucocorticoids, Muscle wasting, Osteoporosis

## Abstract

**Background:**

Patients with rheumatoid arthritis (RA) experience extra-articular manifestations including osteoporosis and muscle wasting, which closely associate with severity of disease. Whilst therapeutic glucocorticoids (GCs) reduce inflammation in RA, their actions on muscle and bone metabolism in the context of chronic inflammation remain unclear. We utilised the TNF-tg model of chronic polyarthritis to ascertain the impact of therapeutic GCs on bone and muscle homeostasis in the context of systemic inflammation.

**Methods:**

TNF-tg and wild-type (WT) animals received either vehicle or the GC corticosterone (100 μg/ml) in drinking water at onset of arthritis. Arthritis severity and clinical parameters were measured, serum collected for ELISA and muscle and bone biopsies collected for μCT, histology and mRNA analysis. In vivo findings were examined in primary cultures of osteoblasts, osteoclasts and myotubes.

**Results:**

TNF-tg mice receiving GCs showed protection from inflammatory bone loss, characterised by a reduction in serum markers of bone resorption, osteoclast numbers and osteoclast activity. In contrast, muscle wasting was markedly increased in WT and TNF-tg animals receiving GCs, independently of inflammation. This was characterised by a reduction in muscle weight and fibre size, and an induction in anti-anabolic and catabolic signalling.

**Conclusions:**

This study demonstrates that when given in early onset chronic polyarthritis, oral GCs partially protect against inflammatory bone loss, but induce marked muscle wasting. These results suggest that in patients with inflammatory arthritis receiving GCs, the development of interventions to manage deleterious side effects in muscle should be prioritised.

**Electronic supplementary material:**

The online version of this article (10.1186/s13075-019-1962-3) contains supplementary material, which is available to authorized users.

## Introduction

Patients with inflammatory arthritis experience extra-articular manifestations, including osteoporosis and muscle wasting, which closely correlate with measures of disease activity [1–4]. Glucocorticoids (GCs) are effective at controlling inflammation in rheumatoid arthritis (RA) and are recommended as an initial line of therapy for the rapid control of disease [5–7]. However, long-term GC use is associated with osteoporosis and systemic muscle wasting, resulting in increased fracture risk and mortality in patients with RA [8–13].

It remains unclear what the effects of GCs on bone and muscle are, when used to treat new onset inflammatory arthritis, in particular, whether the beneficial effects of controlling articular and systemic inflammation on bone and muscle outweigh their direct catabolic actions in these tissues.

Murine models of polyarthritis have proven a powerful tool in examining the pathophysiology of inflammatory diseases, such as RA. The TNF-tg mouse is a murine model of polyarthritis driven by the transgenic overexpression of the pro-inflammatory cytokine TNFα that proved valuable in the initial validation of anti-TNFα biologicals [14]. We have previously shown that this animal model develops systemic bone loss and muscle wasting in a manner consistent with human disease [15, 16]. In this study, we use the TNF-tg model of polyarthritis and wild-type (WT) counterparts to examine the effects of orally administered anti-inflammatory GCs on bone and muscle metabolism in the context of systemic inflammation. We demonstrate that during active polyarthritis, therapeutic GCs are effective at suppressing synovitis, joint destruction and systemic bone loss, but markedly promote systemic muscle wasting.

## Materials and methods

### TNF-transgenic mouse model

Procedures on animals were performed under guidelines by the Animal (Scientific Procedures) Act 1986 in accordance with the project licence (P51102987) and approved by the Birmingham Ethical Review Subcommittee (BERSC). The TNF-tg model of chronic inflammatory polyarthritis, obtained courtesy of Dr. George Kollias (BSRC Fleming, Athens), were maintained on a C57BL/6 background and compared to WT littermates [17]. At day 32 of age, at the first onset of measurable polyarthritis, male TNF-tg mice received drinking water supplemented with either corticosterone (Cort) (100 μg/mL, 0.66% ethanol), or vehicle (0.66% ethanol) for 3 weeks. Interventions were designed to model initial preventative bridging therapeutic glucocorticoid treatments in early onset disease. Following the administration of vehicle and corticosterone, mice were scored twice weekly for clinical scores of disease activity and arthritic paw scores as previously described [18, 19]. Mobility of animals within cages was assessed by measuring numbers of rotations walked by animals in a 3-min period and normalised to rotations per minute to get an activity score. At day 53, serum was collected by cardiac puncture under terminal anaesthesia, and tissues excised for analysis. Wet tissue weights (mg) of tibialis anterior, quadriceps and tibia were recorded, normalised to total body weights and either snap frozen of fixed in 4% formalin (mg).

### Primary human osteoblast culture

Following ethical approval (UK National Research Ethics Committee 14/ES/1044), patients with hip osteoarthritis (OA) (age 69 ± 3 years, Kellgren Lawrence grade 3/4; *n* = 4) were recruited prior to elective joint replacement surgery. Trabecular chips of approximately 400–600 mg were excised and placed in PBS prior to culturing. Reagents were obtained from Sigma (Gillingham, UK) unless otherwise stated. Trabecular bone chips from patient samples were cultured in osteoclast growth media to facilitate release of osteoblasts (Additional file 1: Table S1). Osteoblasts were allowed to grow and once confluent bone chips were removed. Osteoblasts were then differentiated in media containing TNFα (10 ng/ml) and/or cortisol (1000 ng/ml). Treatments were replaced three times per week. Cultures were stained with 0.5% alizarin red S to confirm differentiation into mature osteoblasts.

### Primary human osteoclast culture

Peripheral blood mononuclear cells (PBMCs) from healthy donors, obtained from the Scottish National Blood Transfusion Service (approved by Glasgow NHS Trust-East Ethics Committee), were isolated via Ficoll-paque PLUS (GE Healthcare) density gradient centrifugation and CD14^+^ monocytes isolated using positive selection (Miltenyi). Monocytes were cultured in selective survival media (Additional file 1: Table S1). Osteoclasts were generated using supplementation with1ng/ml RANKL over 72 h before stimulation with vehicle, 10 ng/ml TNFα or 1000 ng/ml corticosterone (or DMSO vehicle) as appropriate. Osteoclast numbers were assessed by staining with tartrate-resistant acid phosphatase (TRAP) kit (Sigma-Aldrich). Osteoclast activity was assessed on mineral-coated plates (Corning) at day 14. Images were acquired using EVOS FL Auto Cell Imaging System (Life Technologies). Osteoclasts were identified as TRAP +ve multinucleated cells (nuclei ≥ 3). Resorption area was calculated using Fiji software (ImageJ) and defined as % resorbed area of entire well.

### Primary murine muscle cell culture

Primary myotubes were generated from tibialis anterior as previously described [20]. In brief, whole tibialis anterior muscle was removed from WT C57/Bl6 animals at 9 weeks and digested in type 1 collagenase at 37 °C for 2 h before isolation of individual fibres. Fibres were plated in 2 ml of muscle expansion medium (Additional file 1: Table S1) and grown in plates coated with Matrigel™ (Corning Life Sciences, Flintshire, UK) (diluted 1/40 in DMEM High Glucose). Satellite cells migrating from muscle fibres were removed and cultured in maintenance medium until confluent, prior to differentiation in selective media for 5 days (Additional file 1: Table S1).

### Gene expression analysis

Gene expression in cells and tissues was assessed using TaqMan® Gene Expression Assays (ThermoFisher Scientific). Tissues were homogenised in liquid nitrogen with a sterile pestle and mortar. mRNA was isolated using an innuPREP RNA Mini Kit (Analytikjena, Cambridge) as per the manufacturer’s instructions. One microgramme of RNA per sample was reverse transcribed using Multiscribe™ using the manufacturer’s protocol (ThermoFisher Scientific) to generate cDNA. Alp, Bglap, Redd1, Foxo1, Trim63 and Fbxo32 were determined using species-specific probe sets by real-time PCR on an ABI7500 system (Applied Biosystems, Warrington, UK). Final reactions are listed in Additional file 1: Table S2. mRNA abundance was normalised to that of 18S or GAPDH. Data were obtained as Ct values and ΔCt determined (Ct target – Ct 18S/GAPDH). Data were expressed as arbitrary units (AU) using the following transformation: [arbitrary units (AU) = 1000 × (2^−Δct^)].

### ELISA analysis

Serum IL-6 (R&D Systems, Abingdon, UK), P1NP and CTX-1 (Immunodiagnostic Systems, Tyne & Wear, UK) and conditioned media pro-collagen I α1 (R&D Systems, Abingdon, UK) were determined using a commercially available ELISA assays in accordance with the manufacturer’s instructions.

### Histological analysis of joints and muscle

Histochemistry was performed on paraffin-embedded 10-μm sections of hind paws and quadriceps of WT and TNF-Tg animals following staining with haematoxylin and eosin. Pannus size at the metatarsal-phalangeal joint interface was determined using Image J software as previously reported [18]. Sections were deparaffinised and incubated in TRAP buffer (Additional file 1: Table S3) for 1 h at 37 °C to detect osteoclasts. Quantification of osteoclast numbers on the bone surface pannus interface of the ulna/humerus joint interface were normalised to bone surface area determined by image J analysis of TRAP-stained paraffin-embedded sections. Sections were stained with H&E prior to quantitative analysis in order to visualise pannus formation at the ankle joints and CSA of fibres. For all quantifications, the mean of data from three adjacent 10-μm sections cut from the centre of the joint or from the vastus medialis from six animals was utilised and assessed using Image J software.

### MicroCT morphometry analysis

Front paws and tibias from mice were imaged using a Skyscan 1172 micro-CT scanner (Bruker) using X-ray beam settings of 60 kV/167 μA with a 0.5-mm aluminium filter. Projections were taken every 0.45° at 580-ms exposure. Image volumes were reconstructed using the Feldkamp algorithm (NRecon 1.6.1.5, Bruker) having applied beam hardening correction. Trabecular bone parameters (bone volume to tissue volume (BV/TV), trabecular thickness (Tb.Th) and trabecular number (Tb.N)) of the tibia were analysed using CTAn software. One millimetre of bone (150 sections) in the metaphyseal region beneath the growth plate was analysed, and regions of interest (ROI) were selected by drawing around the trabecular network for each cross-sectional slice. Front paws were reconstructed, and MeshLab 1.3.2 was used to generate meshes which could then be scored for bone erosions as described previously [18].

### Immunoblot analysis

Briefly, muscle were homogenised in 10-fold volume excess of ice-cold sucrose lysis buffer (Additional file 1: Table S3). Protein concentration was determined using the Bradford protein assay (ThermoScientific). Forty microgrammes of protein was loaded into 4–12% Bis-Tris midi protein gels (Invitrogen) prior to electrophoresis. Proteins were transferred and blocked in blocking buffer (Additional file 1: Table S3) before incubation with primary antibodies (Additional file 1: Table S4) overnight at 4 °C. Membranes were then incubated in horseradish peroxidase-conjugated secondary antibody (1/10,000) at room temperature for 1 h. Antibody detection was performed via enhanced chemiluminescence horseradish peroxidase substrate detection kit (Millipore). Imaging was undertaken using a G:Box Chemi-XR5 (Syngene) and band quantification via (ImageJ). All data were corrected for protein loading as determined after Ponceau S staining (Sigma-Aldrich).

### Statistical analysis

Statistical significance was defined as *P* < 0.05 (*P < 0.05; **P < 0.01; ***P < 0.001) using either an unpaired Student’s *t* test or two way ANOVA with a Bonferroni correction Tukey post hoc analysis where a Gaussian distribution is identified.

## Results

### Oral GCs suppress disease activity in TNF-tg animals

**Fig. 1:**
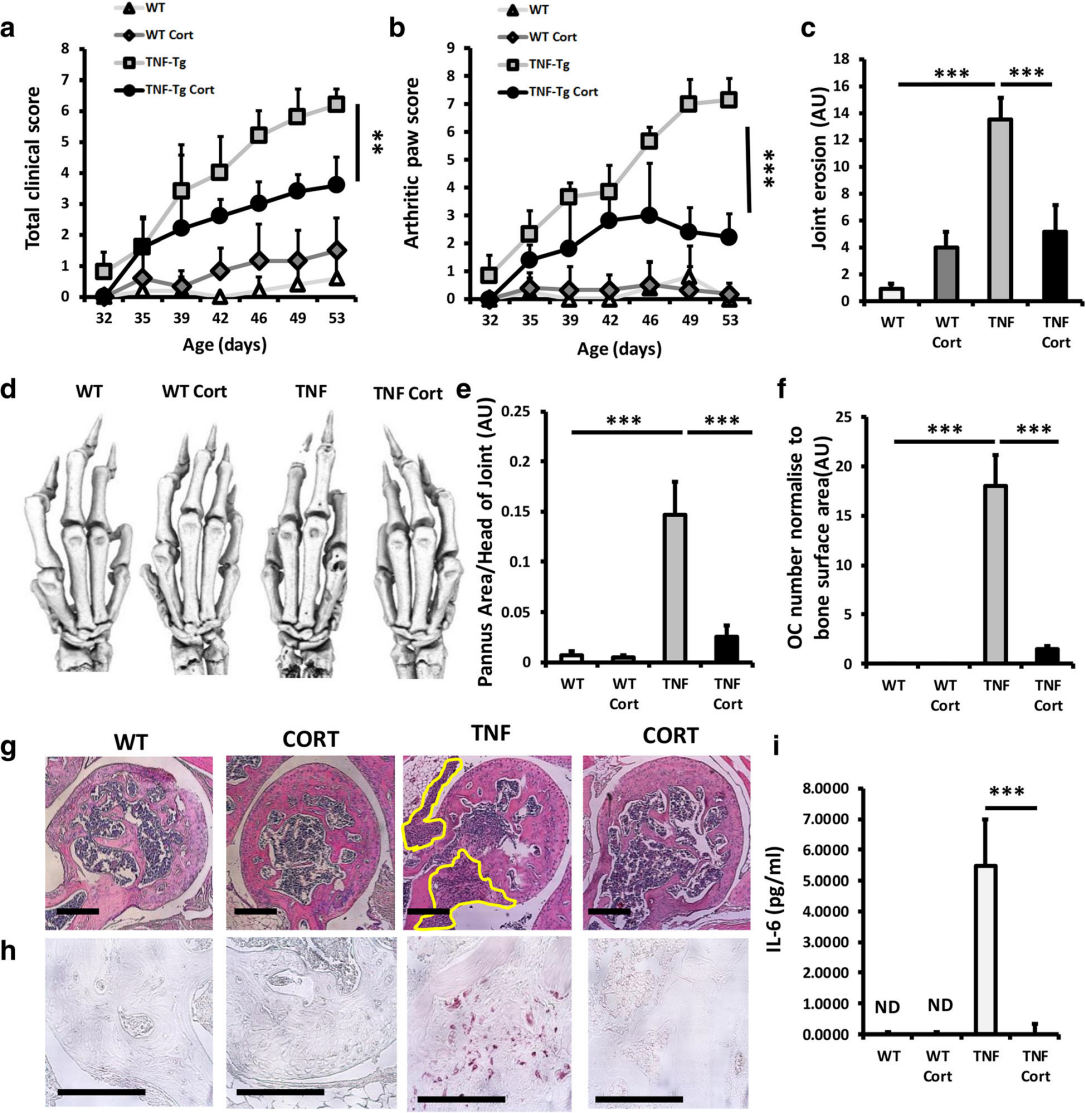
**a** Clinical scoring (weight, inflammation, grimace, behaviour, mobility, inflammation severity and duration); **b** scoring of joint inflammation; **c** quantification of cortical erosion (arbitrary units) in the bones of the ankle, metatarsals and phalanges; **d** representative images of 3D reconstructions of hind paws using micro-CT; **e** histological scoring of synovitis (arbitrary units); **f** histological scoring (arbitrary units) of TRAP +ve osteoclast numbers at the ulna/humerus joint interface; **g** representative images of synovitis at the ulna/humerus joint interface; **h** representative images of TRAP +ve osteoclast numbers at the ulna/humerus joint interface; and **i** serum IL-6 levels determined by ELISA in WT and TNF-tg animals receiving either vehicle or corticosterone (100 μg/mL) in drinking water over 3 weeks. Values are expressed as mean ± standard error of six animals per group. Statistical significance was determined using two-way ANOVA with a Tukey post hoc analysis. Black arrows indicate sites of full-thickness cortical erosions. *P < 0.05, **P < 0.005, ***P < 0.001

TNF-tg mice received drinking water containing vehicle or corticosterone at 100 μg/ml for 3 weeks. Daily oral water intake of corticosterone was calculated per mouse and was shown to be 22.0 ± 0.83 and 23.2 ± 2.0 μg/g body weight/day and did not vary significantly between groups (Additional file 2: Figure S1a). Serum corticosterone was shown to be significantly elevated in both WT and TNF-tg animals receiving corticosterone following oral intake relative to vehicle-treated controls (WT vehicle, 128.3 ± 66.7, WT CORT, 456.2 ± 82.5 ng/ml; *P* < 0.005; TNF-tg vehicle, 119.8 ± 29.9, TNF-tg CORT, 485.1 ± 43.7 ng/ml; *P* < 0.005) (Additional file 2: Figure S1b). Body weights did not vary significantly between groups (Additional file 2: Figure S1c). TNF-tg mice developed significant joint inflammation by day 53, characterised by increased joint deformity, redness and reduced mobility (clinical score, wild type, 0.6 ± 0.004 vs TNF-tg, 6.2 ± 0.3; *P* < 0.005; joint inflammation, wild type, 0.16 ± 0.0001 vs TNF-tg, 7.2 ± 0.7; *P* < 0.0001) (Fig. 1a, b). Corticosterone significantly reduced joint inflammation in TNF-tg animals (clinical score, TNF-tg/vehicle, 6.2 ± 0.3 vs TNF-tg/cort, 3.3 ± 0.9; *P* < 0.005; joint inflammation, TNF-tg/vehicle, 6.2 ± 0.3 vs TNF-tg/cort, 2.2 ± 0.4; *P* < 0.0005) (Fig. 1a, b) [21]. Scoring of synovitis and joint erosions by histology and micro-CT revealed a marked increase in vehicle-treated TNF-tg mice relative to WT controls (Fig. 1c, d, e, g). These were significantly abrogated in TNF-tg animals receiving corticosterone (joint erosion score, TNF-tg/vehicle, 13.6 ± 1.3 vs TNF-tg/cort, 5.3 ± 2.1; *P* < 0.0005, Pannus area, TNF-tg/vehicle, 0.14 ± 3.9 vs TNF-tg/cort, 0.025 ± 0.004; *P* < 0.0005) (Fig. 1c, d, e, g). Juxta articular bone loss was characterised by increased osteoclast numbers at the pannus/subchondral bone interface (Fig 1f, h). Corticosterone treatment reversed this, dramatically reducing osteoclast numbers (TNF-tg/vehicle, 18 ± 3.3 vs TNF-tg/cort, 1.5 ± 0.2; *P* < 0.0005). Serum IL-6 was potently upregulated in vehicle-treated TNF-tg animals and strongly suppressed in animals receiving corticosterone. These data demonstrate that corticosterone administered at 100 μg/ml in drinking water over 3 weeks is sufficient to markedly suppress disease activity and joint destruction.

### Oral GCs prevent trabecular bone loss during polyarthritis

**Fig. 2:**
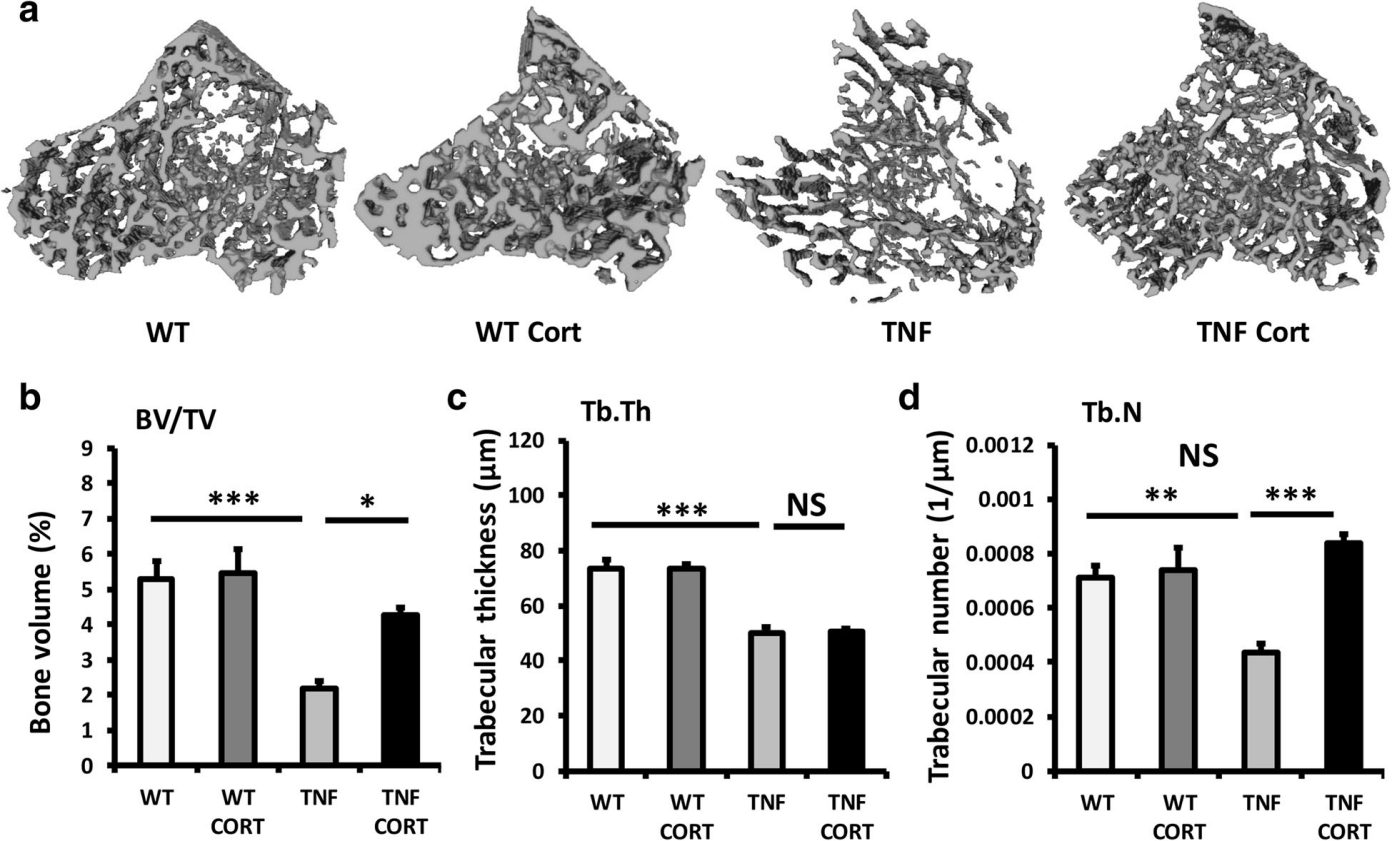
**a** Representative images of 3D reconstructions of tibia trabecular bone using micro-CT, **b** bone volume to tissue volume (BV/TV), **c** trabecular thickness (Tb.Th) and **d** trabecular number (Tb.N) determined by Meshlab software analysis of micro CT in WT and TNF-tg animals receiving either vehicle or corticosterone. Values are expressed as mean ± standard error of six animals per group. Statistical significance was determined using two-way ANOVA with a Tukey post hoc analysis. *P < 0.05, **P < 0.005, ***P < 0.001. Black arrows indicate erosions

In vehicle-treated TNF-tg animals, significant trabecular bone loss was apparent at day 53 (Fig. 2a). This appeared to be partially abrogated in TNF-tg animal receiving corticosterone. Analysis of trabecular bone volume to tissue volume (BV/TV), trabecular thickness (Tb.Th) and trabecular number (Tb.N) was performed in all groups. In vehicle-treated TNF-tg animals, a significant reduction in all parameters was apparent. Treatment with corticosterone partially protected from the loss in BV/TV (BV/TV: TNF-tg/vehicle, 2.1% ± 0.21 vs TNF-tg/corticosterone, 4.3% ± 0.23, *P* < 0.05) (Fig. 2b). Analysis of Tb.Th in TNF-tg animals revealed a similar loss of trabecular thickness in those treated with either vehicle or corticosterone (Tb.Th: TNF-tg/vehicle, 50.2 μm ± 3.7 vs TNF-tg/corticosterone, 50.6 μm ± 2.7, NS) (Fig. 2c). In contrast, corticosterone was able to protect against the reduction in trabecular number in this model of inflammatory polyarthritis (Tb.N: TNF-tg/vehicle, 0.0004 1/μm ± 0.00002 vs TNF-tg/corticosterone, 0.00083 1/μm ± 0.00002, *P* < 0.0001) (Fig. 2d). Together these data demonstrate that oral administration of corticosterone provides partial protection from inflammatory bone loss in TNF-tg mice, characterised by preservation of trabecular number but not thickness.

### GCs suppress both bone formation and resorption during inflammation

**Fig. 3:**
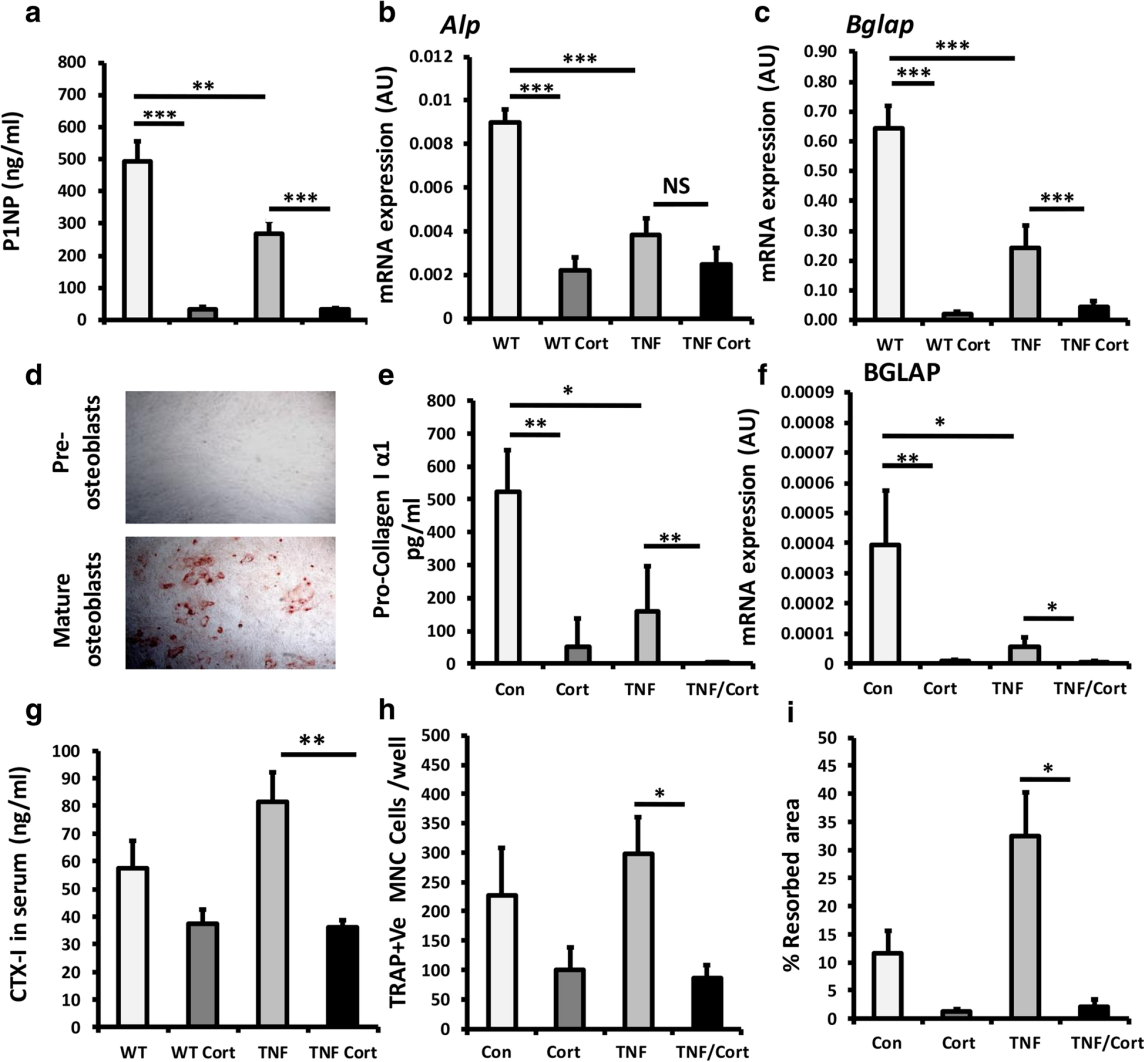
**a** Serum P1NP (ng/ml) determined by ELISA. **b**, **c** Gene expression (AU) of Alp and Bglap, determined by RT qPCR in tibia homogenates, in WT and TNF-tg animals receiving either vehicle or corticosterone (100 μg/mL) in drinking water over 3 weeks. **d** Representative image of primary human pre-osteoblasts and mature nodule forming osteoblasts in vitro stained with alizarin red. **e** Pro-collagen 1 α1 formation determined by ELISA and **f** quantification of gene expression (arbitrary units) of Bglap, determined by RT qPCR in primary cultures of mature osteoblasts treated with either vehicle, TNFα (10 ng/ml), corticosterone (1 μmol/l) or a combination of TNF and corticosterone for 48 h. **g** Serum CTX-1 (ng/ml) determined by ELISA in WT and TNF-tg animals receiving either vehicle or corticosterone. **h** TRAP +ve cells per well and **i** % calcified matrix resorption in primary human osteoclasts cultures treated with either vehicle, TNFα (10 ng/ml), corticosterone (1 μmol/l) or a combination of TNFα and corticosterone over differentiation from mononuclear cells. Values are expressed as mean ± standard error of six animals or primary cultures derived from six separate individuals. Statistical significance was determined using either two-way or one-way ANOVA with Tukey post hoc analysis. *P < 0.05, **P < 0.005, ***P < 0.001

To delineate the actions of corticosterone on bone turnover in TNF-tg mice, we examined systemic markers of bone formation (P1NP) and resorption (CTX-1) and modelled therapeutic GC treatments in human cultures of osteoblasts and osteoclasts in combination with TNFα. Whilst TNF-tg animals had significantly lower P1NP levels at day 53 relative to WT counterparts, both groups developed a comparable suppression of P1NP in response to corticosterone (wild type/vehicle, 494 ng/ml ± 46.3 vs wild type/corticosterone, 31.3 ng/ml ± 8.2; *P* < 0.0001, TNF-tg/vehicle, 269.7 ng/ml ± 27.2 vs TNF-tg/corticosterone, 32.3 ng/ml ± 7.5; *P* < 0.0001) (Fig. 3a). Analysis of mature osteoblast markers in tibia homogenates supported these data. Here, whilst gene expression of alkaline phosphatase (Alp) and osteocalcin (Bglap) were significantly reduced in TNF-tg animals at day 53 relative to WT counterparts (Alp, 2.2-fold; Bglap, 2.6-fold; *P* < 0.0001), a comparable suppression of gene expression was apparent in both groups receiving corticosterone relative to vehicle (wild type, 32-fold; *P* < 0.0001, TNF-tg, 6-fold; *P* < 0.0001) (Fig. 3b, c). In mature primary human osteoblasts, incubation with the pro-inflammatory cytokine TNFα resulted in a significant reduction in both pro-collagen production and osteocalcin mRNA (Fig. 3d, f). Here, the addition of the GC cortisol resulted in a comparable and dramatic suppression of osteoblast matrix formation and mRNA expression in control and TNFα-treated osteoblasts (pro-collagen Iα1, control, 524 ng/ml ± 128.9 vs cortisol, 50.0 ng/ml ± 93.1, *P* < 0.001, TNFα, 158.2 ng/ml ± 131.4 vs TNFα/cortisol, 11.3 ng/ml ± 6.8; *P* < 0.001; BGLAP, control vs cortisol, 43-fold suppression; *P* < 0.001, TNFα vs TNFα/cortisol; 10-fold suppression; *P* < 0.05).

Serum CTX-1 levels were determined as a measure of osteoclastic bone resorption. TNF-tg animals receiving corticosterone had a significant suppression of CTX-1 at day 53 (TNF-tg/vehicle, 87.6 ng/ml ± 11.3 vs TNF-tg/corticosterone, 36.3 ng/ml ± 3.1; *P* < 0.05) (Fig. 3g). In primary human osteoclasts, the addition of cortisol to TNFα-stimulated cultures resulted in a significant suppression of both osteoclast numbers and calcified matrix resorption in vitro (osteoclast no., TNFα, 297.5 cells per well ± 53.7 vs TNFα/cortisol, 85.6 cells per well ± 17.8; *P* < 0.05; resorbed area, TNFα, 32.5% ± 7.0 vs TNFα/cortisol, 2.1% ± 0.8; *P* < 0.05) (Fig. 3h, i). The resorption pits in wells treated with cortisol were characterised by a reduction in both number and size (Additional file 3: Figure S2a). These data indicate that GCs suppress both osteoblast bone formation and osteoclast maturation and activity.

### Oral GCs drive severe muscle wasting and reduce mobility in TNF-tg animals

**Fig. 4:**
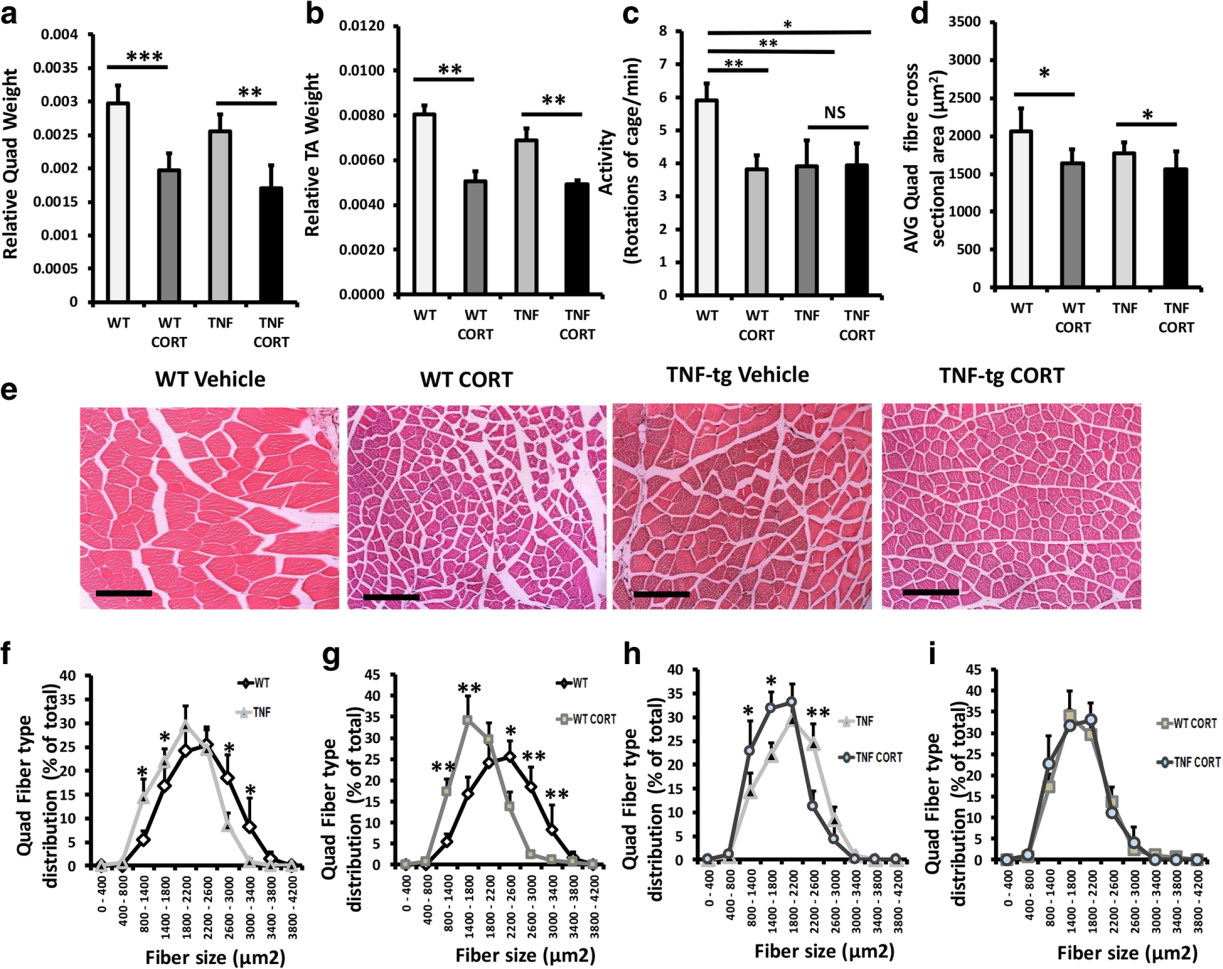
**a**, **b** Total quadriceps and tibialis anterior muscle weights relative to total bodyweight, **c** mouse activity determined by rotations of cage per minute, **d** average quadriceps muscle fibre cross sectional area (μm^2^), **e** representative images of quadriceps muscle sections, **f**–**i** distribution of quadriceps muscle fibre cross-sectional area determined using Image J in paraffin embedded sections in WT and TNF-Tg animals receiving either vehicle or corticosterone (100 μg/mL) in drinking water over 3 weeks. Values are expressed as mean ± standard error of six animals per group. Statistical significance was determined using two-way ANOVA with a Tukey post hoc analysis. *P < 0.05, **P < 0.005, ***P < 0.001 (scale bars, 50 μm)

We examined muscle weights and morphology in WT and TNF-tg animals receiving corticosterone. Corticosterone significantly reduced quadriceps and tibialis anterior weights in WT and TNF-tg animals relative to vehicle controls (quadriceps, wild type/vehicle, 0.0029 mg/body weight ± 0.00024 vs wild type/corticosterone, 0.0019 mg/body weight ± 0.00021; *P* < 0.0001; TNF-tg/vehicle, 0.0025.7 mg/body weight ± 0.00025 vs TNF-tg/corticosterone, 0.0017 mg/body weight ± 0.00028; *P* < 0.001) (Fig. 4a, b). Analysis of animal mobility with cages was assessed at day 53 to determine the effects of polyarthritis and corticosterone treatment (Fig. 4c). Here, a significant reduction in movement was apparent in TNF-tg animals relative to WT counterparts. This was mirrored by a comparable reduction in movement seen in both WT and TNF-tg animals receiving corticosterone relative to vehicle-treated WT counterparts. Analysis of average muscle fibre cross-sectional area (CSA) indicated that this was underpinned by a reduction in muscle fibre size in WT and TNF-tg animals receiving corticosterone (fibre size, wild type/vehicle, 2064 μm^2^ ± 144 vs wild type/corticosterone, 1636 μm^2^ ± 96; *P* < 0.05; TNF-tg/vehicle, 1767 μm^2^ ± 76 vs TNF-tg/corticosterone, 1559 μm^2^ ± 88; *P* < 0.05) (Fig. 4c–e). Further analysis of fibre CSA distribution identified a significant shift towards increased small diameter fibres in TNF-tg animals relative to WT counterparts (Fig. 4e). In response to corticosterone, both WT and TNF-tg animals demonstrated a further shift in fibre CSA distribution, favouring a significant increase in small fibres (800–1800 μm^2^) and significant reduction in large fibres (2200–2600 μm^2^) relative to vehicle-treated controls (Fig. 4f, g). In contrast, no significant shift was observed between WT and TNF-tg animals receiving corticosterone (Fig 4h). These data demonstrate that during active polyarthritis, administration of oral corticosterone promotes muscle wasting independent of inflammation.

### During inflammation, oral GCs drive catabolic and anti-anabolic muscle wasting

**Fig. 5:**
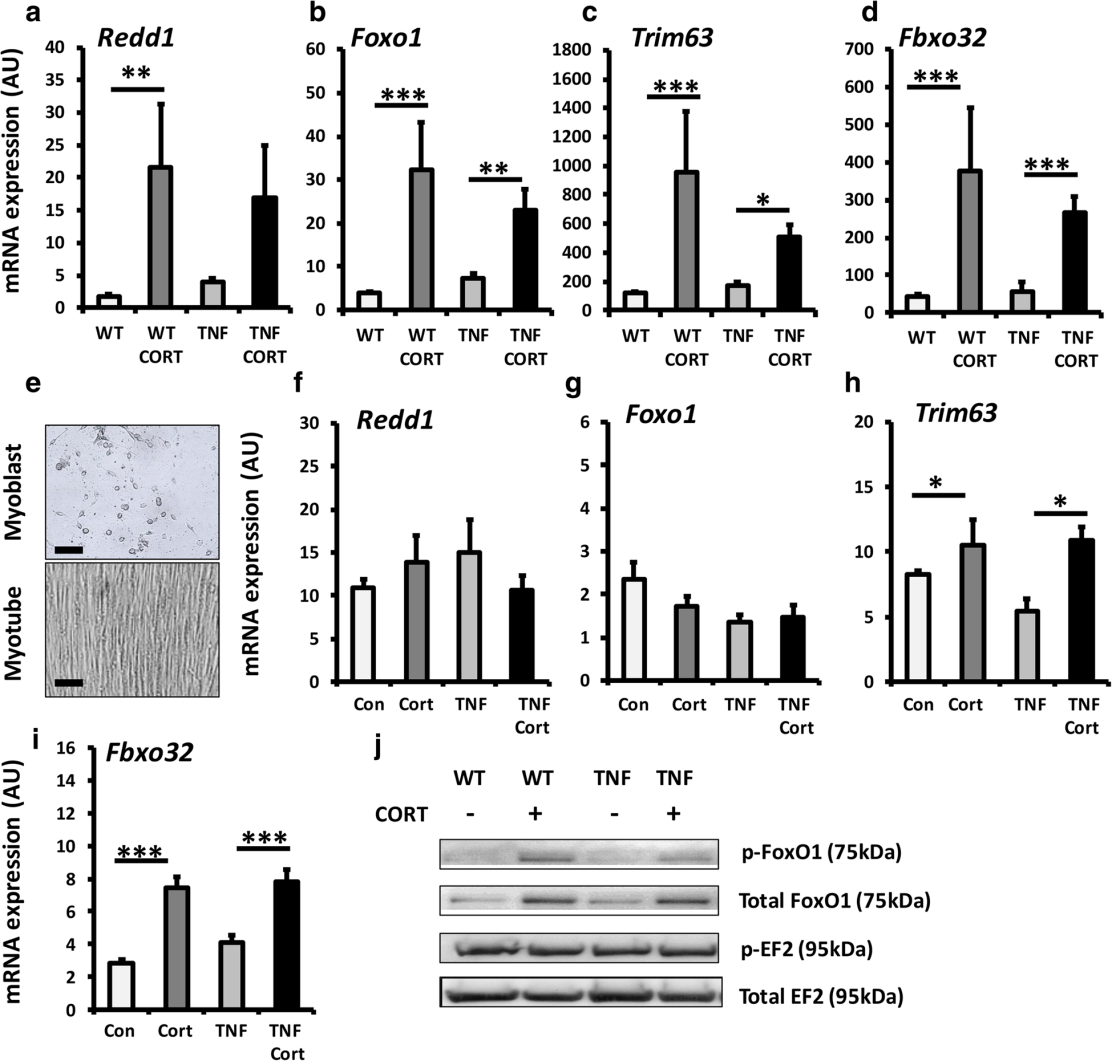
**a**–**d** Quantification of gene expression (arbitrary units) of Redd1, Foxo1, Trim63 and Fbxo32, determined by RT qPCR, in ex vivo quadriceps biopsies. **e**–**h** Representative images of primary murine myoblast and myotubes and quantification of gene expression of Redd1, Foxo1, Trim63 and Fbxo32, determined by RT qPCR and **j** representative western blot staining for p-Foxo1, total Foxo1, pEF2 and p-EF2 after loading of 20 μg of protein and normalisation to ponso staining in quadriceps for either WT mice and TNF-Tg animals receiving either vehicle or corticosterone (100 μg/mL) in drinking water over 3 weeks or in primary myotubes treated with either vehicle, TNFα (10 ng/ml), corticosterone (1 μmol/l) or a combination of TNF and corticosterone for 48 h. Values are expressed as mean ± standard error of six animals or primary cultures derived from four separate animals. Statistical significance was determined using two-way ANOVA with a Tukey post hoc analysis. *P < 0.05, **P < 0.005, ***P < 0.001

To ascertain the pathways that underpin increased muscle wasting in TNF-tg mice treated with GCs, we examined well-defined catabolic and anti-anabolic signalling pathways in tibialis anterior muscle homogenates and in primary muscle cultures. In wild-type animals, corticosterone resulted in a comparable induction in expression of the anti-anabolic gene Redd1 and the catabolic genes Foxo1, Trim63 and Fbxo32 (Redd1, 7.7-fold; *P* < 0.005; Foxo1, 10.3-fold; *P* < 0.0005; Trim63, 7.8-fold; *P* < 0.05; Fbxo32, 8.9-fold; *P* < 0.0005) (Fig. 5a–d). Comparable inductions in gene expression of Foxo1, Trim63 and Fxo32 were also apparent in TNF-tg animals receiving corticosterone (Foxo1, 3.1; *P* < 0.005, Trim63, 3.2-fold; *P* < 0.05; Fbxo32, 5.2-fold; *P* < 0.0005) (Fig. 5b–d, Additional file 4: Figure S3). These results were supported by a marked increase in protein expression of both phosphorylated and total Foxo1 in WT and TNF-tg animals in response to corticosterone (Fig. 5j). In contrast, expression of anabolic factors such as EF2 did not differ between groups. A deeper analysis of muscle gene expression was performed examining catabolic signalling and E3 ligases (*Foxo1, Fbxo32, Trim63, Ube3a*), anabolic and anti-anabolic myokines and signalling (*Igf1, Igf2, Mstn, Redd1*), muscle differentiation (*Myog, MyoD, Myf5, Myf6*) and inflammatory myokines and signalling (*Tnfa, Il6, Cxcl1, IkBa*) (Additional file 3: Figure S2a-p). In addition to the upregulation of all atrogenes examined, gene expression of the inflammatory myokines *Il6* and *Cxcl1* showed evidence of suppression in response to corticosterone in line with expected anti-inflammatory action. Similar observations were apparent in primary cultures of murine muscle cells treated with corticosterone. Here, a significant upregulation was observed in Trim63 and Fbxo32 regardless of inflammatory stimulation with TNFα (Fig. 5e–i). These data demonstrate that at therapeutic doses, GCs result in a significant induction in anti-anabolic and catabolic gene expression in muscle.

## Discussion

We employed a murine model of polyarthritis treated with oral corticosterone to examine the effects of GCs on bone and muscle in the context of initial preventative bridging therapy in early-onset polyarthritis. This approach was effective at suppressing disease activity and joint destruction and has previously been demonstrated to mimic the kinetics of oral GC therapy [22]. Clinically, GCs suppress disease activity, joint destruction and systemic inflammation, which are drivers of bone and muscle loss in RA [1, 23–26]. However, whilst effective in controlling disease activity, they are independently associated with GC-induced osteoporosis and muscle wasting through direct anti-anabolic and catabolic pathways [8–13, 27, 28]. Currently, their impact on bone and muscle when used to treat new-onset inflammatory arthritis remains unclear.

The anti-inflammatory properties of oral GC administration were evidenced by a marked suppression in disease activity, joint inflammation and joint destruction in the TNF-tg model. We observed a marked decrease in trabecular bone volume at day 53 in TNF-tg mice, characterised by an increase in osteoclast activity and numbers. Administration of oral GCs resulted in a significant protection from this inflammatory bone loss.

Increased osteoclast numbers and activity are recognised mediators of inflammatory bone loss in RA, whilst therapeutic control of inflammation abrogates this [29–31]. Our study mirrors these findings, where TRAP +ve osteoclast numbers and bone resorption determined by serum CTX1 were significantly reduced in animals receiving corticosterone. In vitro data supported these findings where osteoclast numbers and activity were markedly reduced by GCs in TNFα-stimulated osteoclasts. These data indicate that the protection from inflammatory bone loss in TNF-tg animals receiving corticosterone are mediated through the suppression of osteoclastic bone resorption.

Both inflammation and GCs are reported to be negative regulators of bone formation and osteoblastogenesis [32–34]. Here, the anti-anabolic actions of GCs on bone formation are associated with a rapid suppression of P1NP and reduction in trabecular bone in patients [34].

In our study, whilst markers of mature osteoblasts and bone formation were suppressed in TNF-tg animals, therapeutic GCs dramatically exacerbated this and mirror observations in patient studies. These data suggest that the direct anti-anabolic actions of GCs outweigh positive effects of suppressing inflammation on bone formation in vivo.

Several studies have explored the effects of therapeutic GCs on bone metabolism in RA. These yielded conflicting outcomes and are complicated by differences in disease severity, parallel DMARD therapy and dosing of GCs. Several reported increased fracture risk, with complications more apparent at higher doses [23, 35–37]. Others reported no worsening of bone mineral density or fracture risk when given at low doses in combination with DMARDS [3, 38, 39].

In contrast to bone loss, oral GCs increased muscle wasting and did not restore animal mobility in the TNF-tg model of polyarthritis, despite effective suppression of disease activity. This was characterised by a comparable decrease in muscle wet weights and fibre size in WT and TNF-tg animals suggesting that these changes occur independent of inflammatory muscle wasting and that GC-driven muscle wasting impacts on animal mobility.

In patients receiving therapeutic GCs, muscle wasting is characterised by increased protein breakdown driven through the ubiquitin proteasome and the lysosomal systems [11–13, 28]. Our results mirror this with an upregulation of anti-anabolic and catabolic pathway activation in animals receiving corticosterone.

Similar results were observed in primary muscle cultures, where regardless of pro-inflammatory stimulation with TNFα, a direct induction of catabolic gene expression was observed in response to GCs. Additive or synergistic catabolic actions in muscle by oral GCs in combination with inflammation were not observed in this study. This is most likely attributed to the effective suppression of well-defined inflammatory mediators of muscle wasting that are themselves suppressed by GCs [40–42].

Few studies address therapeutic GC use on muscle wasting in RA. However, of note, a recent study by Lemmey et al. reported a rapid loss of muscle mass in RA patients receiving a single intramuscular injection of GCs to treat disease flares [43]. Unfortunately, this study was not able to address the impact of disease activity (itself a significant driver of muscle wasting) and DMARD use on muscle wasting independent of corticosterone use. However, our study supports the author’s conclusions that muscle wasting is an immediate and severe complication in RA patients receiving GCs, independent of inflammation. Indeed, the marked increase in fracture risk upon initiation of GCs in RA may be primarily driven by GC-induced muscle wasting, rather than secondary to GC-induced osteoporosis [44].

## Conclusions

Using an animal model of chronic polyarthritis, this study examined how controlling disease activity with oral GCs as a monotherapy influenced inflammatory osteoporosis and muscle wasting. We demonstrate that when given in early disease, oral GCs protect against inflammatory bone loss, but induce marked systemic muscle wasting. These results suggest that the development of interventions to manage deleterious side effects in muscle should be prioritised in patients with inflammatory arthritis receiving GCs.

## Additional files

Additional file 1: **Table S1.** Media for primary culture. **Tables S2.** Real-time PCR Master mix. **Table S3.** Buffers. **Table S4.** IgGs used in Immunoblotting. (DOCX 17 kb)

Additional file 2: **Figure S1.** (a) body weights (g), (b) daily corticosterone intake (μg/g body weight/day) and (c) serum corticosterone determined by ELISA (ng/ml) in in WT and TNF-Tg animals receiving either vehicle or corticosterone (100 μg/mL) in drinking water over 3 weeks. Values are expressed as mean ± standard error of six per group for weight and at least three animals per group for steroid intake and serum measurement. Statistical significance was determined using two-way ANOVA with a Tukey post hoc analysis. * *P* < 0.05, **P < 0.005, ***P < 0.001. (TIF 435 kb)

Additional file 3: **Figure S2.** Representative images of human primary culture osteoclast activity assessed on mineral-coated plates at day 14 treated with vehicle, cortisol (1000 nmol/l), TNFa 10 ng/ml) or a combination of both. Images were acquired using EVOS FL Auto Cell Imaging System (Life Technologies). (TIF 1090 kb)

Additional file 4: **Figure S3.** (a-p) Gene expression of *Foxo1*, *Fbxo32*, *Trim63*, *Ube3a*, *Igf1*, *Igf2*, *Mstn*, *Redd1*, *Myog*, *MyoD*, *Myf5*, *Myf6*, *Tnfa*, *Il6*, *Cxcl1* and *IkBa* were determined by RT qPCR in quadriceps for either WT mice and TNF-Tg animals receiving either vehicle or corticosterone (100 μg/mL) in drinking water over 3 weeks. Values are expressed as mean ± standard error of six animals or primary cultures derived from four separate animals. Statistical significance was determined using two-way ANOVA with a Tukey post hoc analysis. *P < 0.05, **P < 0.005, ***P < 0.001. (TIF 1454 kb)

## Data Availability

All data generated or analysed during this study are included in this published article [and its supplementary information files].
