# Supplementary material for: Therapeutic glucocorticoids prevent bone loss but drive muscle wasting when administered in chronic polyarthritis
Source: Arthritis Res Ther. 2019 Aug 1;21:182. doi: 10.1186/s13075-019-1962-3 (PMC6676537; doi:10.1186/s13075-019-1962-3)
Supplement: Supplementary file 1 — Table S1. Media for primary culture. Tables S2. Real-time PCR Master mix. Table S3. Buffers. Table S4. IgGs used in Immunoblotting. (DOCX 17 kb) [file 13075_2019_1962_MOESM1_ESM.docx]

Supplementary Table

| **Primary culture media** | **Media** | **Supplements** |
| --- | --- | --- |
| Human Osteoblast Culture Media | High glucose Dulbecco’s Modified Eagle Medium (DMEM) | 10 % fetal calf serum,  1 % sodium pyruvate,  1 % non-essential amino acids,  1 % penicillin-streptomycin,  50 μg/ml ascorbic acid  2 mM β-glycerophosphate |
| Human Osteoclast Culture Media | α-MEM | 10 % fetal calf serum,  0.02 mM L-glutamine,  1 % penicillin-streptomycin,  25 ng/ml M-CSF (PeproTech) |
| Murine Muscle Expansion Media | High glucose Dulbecco’s Modified Eagle Medium (DMEM) | 30 % fetal calf serum,  10 % horse serum  1 % Chick Embryo Extract  10 ng/ml basic fibroblast growth factor (PeproTech) |
| Murine Muscle Maintenance Media | High glucose Dulbecco’s Modified Eagle Medium (DMEM) | 10 % horse serum,  0.5 % Chick Embryo Extract |
| Muscle Differentiation Media | High glucose Dulbecco’s Modified Eagle Medium (DMEM) | 2 % horse serum |

Table 1: Media for primary culture

| real-time PCR Master mix |  |
| --- | --- |
| 2X TaqMan PCR mastermix (Life Technologies), | 5 ul |
| 200 nmol TaqMan probe | 0.5 ul |
| RNAse free water | 3.5 ul |
| cDNA | 25–50 ng in 1 ul |

Table 2: real-time PCR Master mix

| Buffers |  |
| --- | --- |
| Sucrose Lysis buffer | 50 mM Tris/HCl (pH 7.5),  250 mM Sucrose  1 mM EGTA  50 mM NaF  10 mM Na-β-Glycerophosphate,  1 mM Benazmidine  1 % Triton X-100  0.1 % β-Mercaptoethanol  5 mM Na-Pyrophosphate,  1 mM EDTA  1 mM Na3VO4  supplemented with protease inhibitor cocktail |
| Blocking buffer, Tris-buffered saline Tween-20 | 0.137 M NaCl  0.02 M Tris-base 7.5pH  0.1 % Tween-20  5 % skimmed milk in (TBS-T, 0.137M |
| TRAP buffer (pH 5) | 0.1 M sodium acetate,  75 mM L+ tartaric acid,  1.6 mM Fast Red Violet LB salt  0.5 % naphthol AS-MX phosphate |
|  |  |
|  |  |

Table 3: Buffers

| **IgG** | **Manufacturer** | **Cat No** | **Diln** |
| --- | --- | --- | --- |
| FoxO1/FoxO3a T24/T32 | Cell Signaling Technology | cat no: #9464, | (1/500), |
| p-FoxO1/FoxO3a | Cell Signaling Technology | cat no: #2880 | (1/1000), |
| eEF2 | Cell Signaling Technology | cat no: #2332 | (1/5000), |
| p-eEF2 T56 | Cell Signaling Technology | cat no: # 2331 | (1/5000) |

Table 4. IgGs used in Immunoblotting
