# Supplementary figures and images for: Therapeutic glucocorticoids prevent bone loss but drive muscle wasting when administered in chronic polyarthritis
Source: Arthritis Res Ther. 2019 Aug 1;21:182. doi: 10.1186/s13075-019-1962-3 (PMC6676537; doi:10.1186/s13075-019-1962-3)

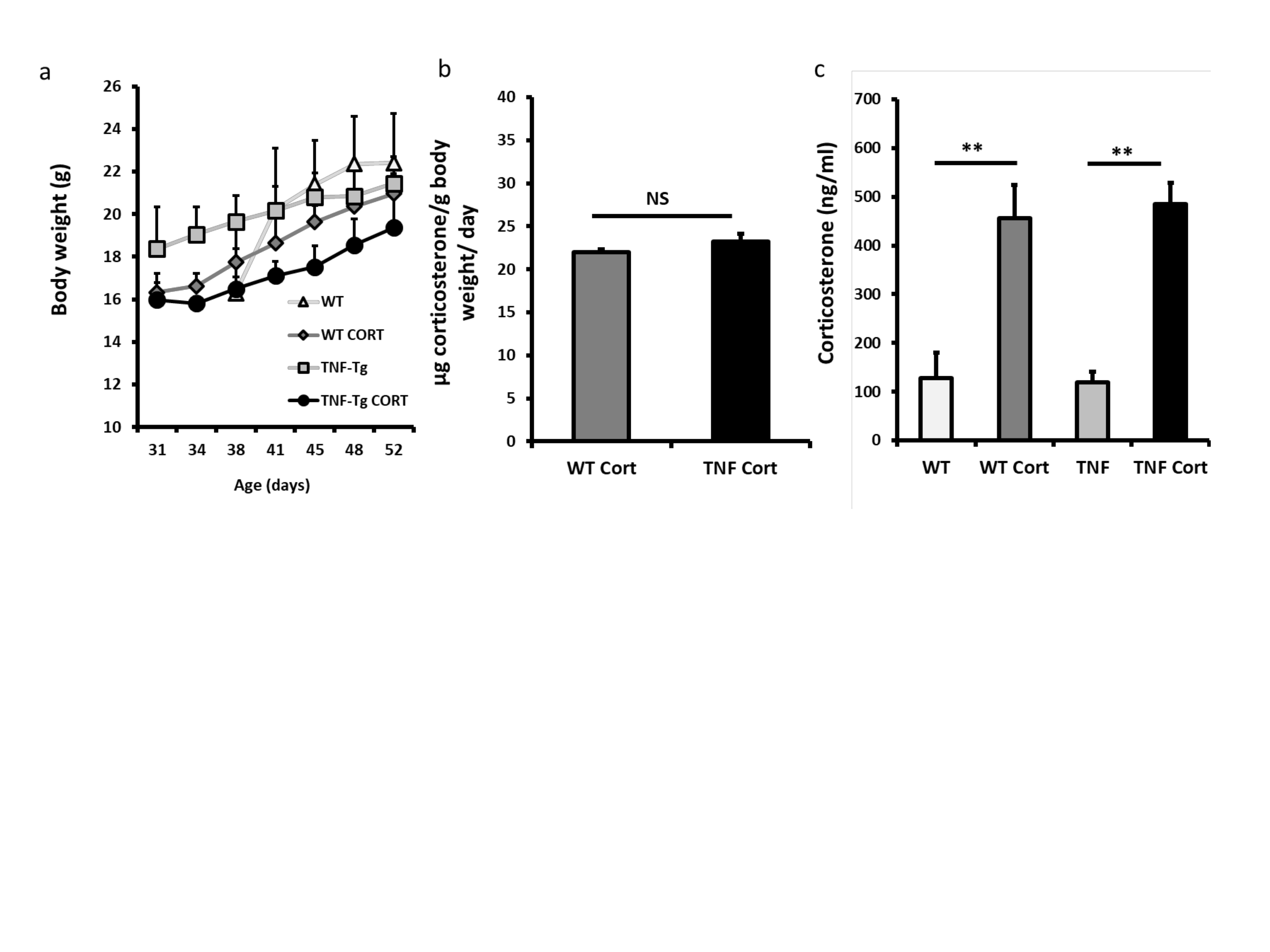

Supplement: Supplementary file 2 — Figure S1. (a) body weights (g), (b) daily corticosterone intake (μg/g body weight/day) and (c) serum corticosterone determined by ELISA (ng/ml) in in WT and TNF-Tg animals receiving either vehicle or corticosterone (100 μg/mL) in drinking water over 3 weeks. Values are expressed as mean ± standard error of six per group for weight and at least three animals per group for steroid intake and serum measurement. Statistical significance was determined using two-way ANOVA with a Tukey post hoc analysis. * P < 0.05, **P < 0.005, ***P < 0.001. (TIF 435 kb) [file 13075_2019_1962_MOESM2_ESM.tif]

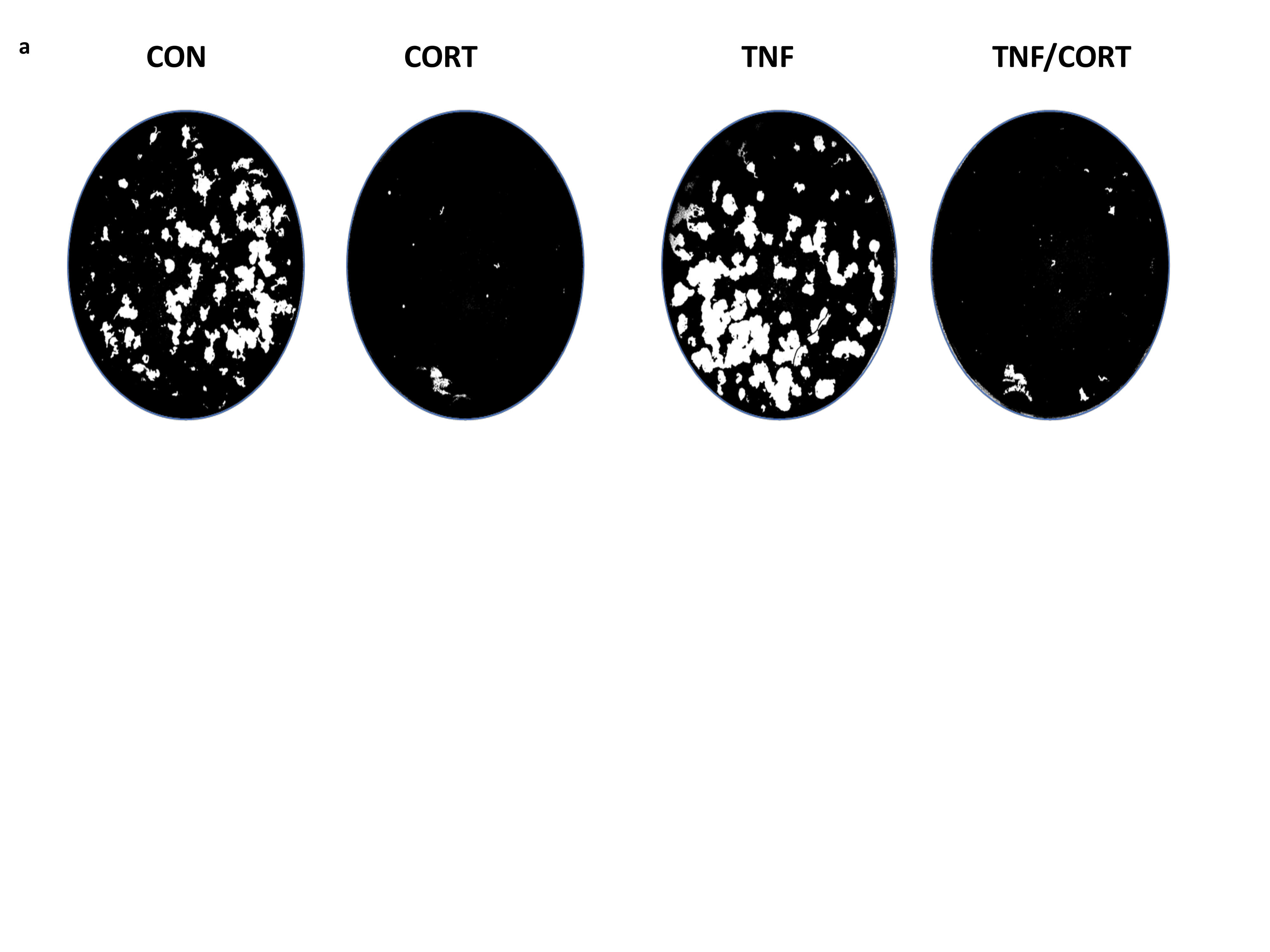

Supplement: Supplementary file 3 — Figure S2. Representative images of human primary culture osteoclast activity assessed on mineral-coated plates at day 14 treated with vehicle, cortisol (1000 nmol/l), TNFa 10 ng/ml) or a combination of both. Images were acquired using EVOS FL Auto Cell Imaging System (Life Technologies). (TIF 1090 kb) [file 13075_2019_1962_MOESM3_ESM.tif]

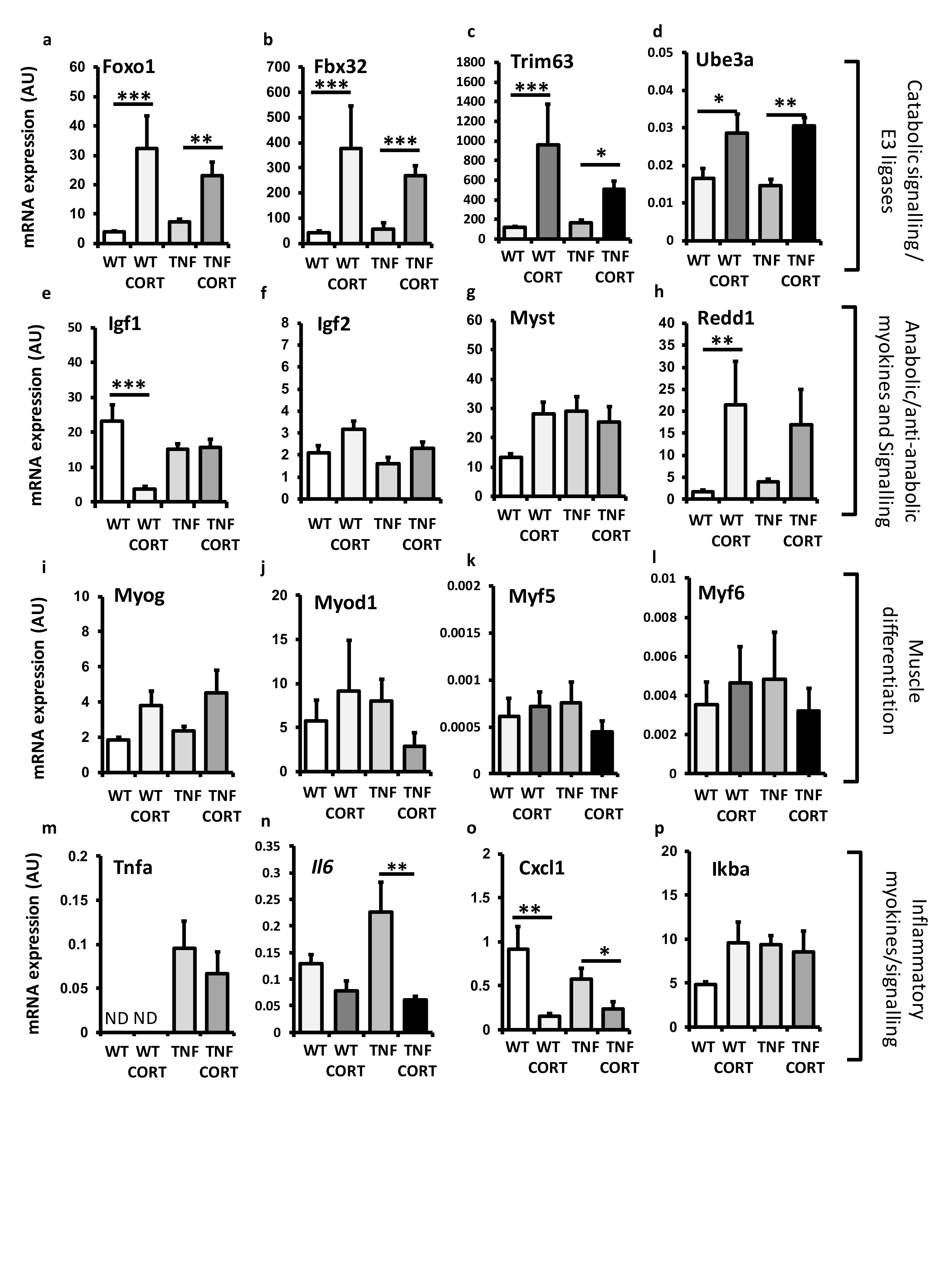

Supplement: Supplementary file 4 — Figure S3. (a-p) Gene expression of Foxo1, Fbxo32, Trim63, Ube3a, Igf1, Igf2, Mstn, Redd1, Myog, MyoD, Myf5, Myf6, Tnfa, Il6, Cxcl1 and IkBa were determined by RT qPCR in quadriceps for either WT mice and TNF-Tg animals receiving either vehicle or corticosterone (100 μg/mL) in drinking water over 3 weeks. Values are expressed as mean ± standard error of six animals or primary cultures derived from four separate animals. Statistical significance was determined using two-way ANOVA with a Tukey post hoc analysis. *P < 0.05, **P < 0.005, ***P < 0.001. (TIF 1454 kb) [file 13075_2019_1962_MOESM4_ESM.tif]
